# Supplementary material for: Modelling the impact of changes to abdominal aortic aneurysm screening and treatment services in England during the COVID-19 pandemic
Source: PLoS One. 2021 Jun 15;16(6):e0253327. doi: 10.1371/journal.pone.0253327 (PMC8205127; doi:10.1371/journal.pone.0253327)
Supplement: S1 Table — (DOCX) [file pone.0253327.s001.docx]

# S1 Table. Input parameters used in the discrete event simulation model

| Parameter | **Estimate** | **Source** |
| --- | --- | --- |
| Screening |  |  |
| Re-invitation | 0.1360 | MASS (1) |
| Attendance proportion | 0.750 | NAAASP (2015/16) |
| Non-visualisation proportion | 0.0121 | MASS |
| Age and AAA size distribution at baseline  Invited cohort^§^  Surveillance cohort | 65-year old, AAA distribution obtained from first 700,000 men screened  Age and AAA distribution obtained from NAAASP surveillance cohort | NAAASP (2009-2014)(2)  NAAASP (May 2020) |
| AAA growth *^§^ | Mean growth rates:  1.8mm/yr for 3.0cm AAA  2.3mm/yr for 4.0cm AAA  2.9mm/yr for 5.0cm AAA | MASS |
| AAA rupture †^§^ | 3.0cm AAA: 0.03 per 100 p-years  4.0cm AAA: 0.17 per 100 p-years  5.0cm AAA: 0.64 per 100 p-years  5.5cm AAA: 1.13 per 100 p-years | RESCAN (3) (11 studies) that record rupture rates for men |
| Surveillance |  |  |
| Dropout rate | 5.72 per 100 p-years | MASS |
| Incidental detection rate | 4.59 per 100 p-years | Glover et al.(4) |
| Delay from 5.5+cm scan to consultation | 71 days | MASS |
| Consultation scan | CT diameter:  Mean = US + 0.244cm, SD 0.19cm | RESCAN & Singh et al.(5) |
| Non-intervention proportion | 0.125 | MASS |
| Delay from consultation to surgery | 59 days | MASS |
| Elective operations |  |  |
| Proportion receiving EVAR vs. Open | 0.74 at age 80, AAA diameter 6.0cm. Odds ratio 1.10 per year increase in age, 0.74 per cm increase in diameter | National Vascular Registry(6) |
| Elective EVAR 30-day mortality | 0.008 at age 80, AAA diameter 6.0cm. Odds ratio 1.10 per year increase in age, 1.33 per cm increase in diameter | National Vascular Registry |
| Elective Open 30-day mortality | 0.051 at age 80, AAA diameter 6.0cm. Odds ratio 1.09 per year increase in age, 1.12 per cm increase in diameter. | National Vascular Registry |
| Re-intervention rate after successful elective EVAR | 13.5 and 3.6 per 100 person-years during 31-120 and >120 days respectively | EVAR-1(7) |
| Re-intervention rate after successful elective open repair | 1.6 and 1.3 per 100 person-years during 31-120 and >120 days respectively | EVAR-1 |
| Long-term AAA mortality after elective EVAR | 0.8 per 100 person-years | EVAR-1 |
| Long-term AAA mortality after elective Open | 0.07 per 100 person-years | EVAR-1 |
| Emergency operations |  |  |
| Proportion operated after rupture | 0.368 | MASS |
| Proportion receiving EVAR vs. Open | 0.22 at age 80. Odds ratio 1.05 per year increase in age | National Vascular Registry |
| Emergency EVAR 30-day mortality | 0.22 at age 80. Odds ratio 1.05 per year increase in age | National Vascular Registry |
| Emergency Open 30-day mortality | 0.44 at age 80. Odds ratio 1.07 per year increase in age | National Vascular Registry |
| Re-intervention rate after successful emergency EVAR | 10.9 per 100 person-years | IMPROVE(8, 9) |
| Re-intervention rate after successful emergency open repair | 6.1 per 100 person-years | IMPROVE |
| Long-term AAA mortality after emergency EVAR | 1.0 per 100 person-years | IMPROVE |
| Long-term AAA mortality after emergency open repair | 1.4 per 100 person-years | IMPROVE |
| Miscellaneous |  |  |
| Non-AAA mortality rate | UK population age/sex specific | Office for National Statistics(10) |
| QoL utilities | Annual utilities from 0.81 at age 65, 0.77 at age 75, 0.74 at age 85 | Love-Koh et al.(9) |
| Discounting rates | 3.5% per year for life-years and costs |  |

MASS – Multicentre Aneurysm Screening Study

NAAASP – National Abdominal Aortic Aneurysm Screening Programme

**^§^** Assumed the same for non-attenders

* Longitudinal linear mixed model for log AAA diameter: Slope ($\beta_{1}=0.058$), Intercept ($\beta_{0}=1.27$), Slope log SD ($\log(\sigma_{1})=-3.32$), Intercept log SD ($\log\left( \sigma_{0} \right)=-1.74$), Arctanh correlation ($\mathrm{atanh} (\rho)=0.46$), Residual log SD ($\log(\sigma_{w})=-2.59$)

** $N(\mu,\Sigma)$ where $\mu=\left( \begin{matrix} 0.058 & 1.27 & -3.32 & -1.74 & 0.46 & -2.59 \end{matrix} \right)$, and

$$\Sigma=\left( \begin{matrix} 2.0\times{10}^{-6} & & & & & \\ 1.7{\times10}^{-6} & 0.000030 & & & & \\ 0 & 0 & 0.001714 & & & \\ 0 & 0 & -0.000019 & 0.000528 & & \\ 0 & 0 & 0.000483 & 0.000048 & 0.002588 & \\ 0 & 0 & -0.000068 & -1.4\times{10}^{-6} & 9.3\times{10}^{-6} & 0.000081 \end{matrix} \right)$$

† Data for rupture rates obtained from 11 RESCAN studies (Western Australia, Chichester, Gloucestershire, Huntingdon, MASS, Manchester, Tromso, Galdakao, Stirling, UKSAT, Viborg). See eTable 2 of (3) for further information on these studies. Joint model for log rupture rates and log underlying AAA diameter were fitted to each study separately then combined using multivariate meta-analysis: association with diameter ($\gamma_{1}=5.92$), Intercept ($\gamma_{0}=-14.57)$

‡ $N(\mu,\Sigma)$ where $\mu=\left( 5.92, -14.57 \right)$, and $\Sigma=\left( \begin{matrix} 0.8282 & -1.1190 \\ -1.1190 & 1.5391 \end{matrix} \right)$

# References

1. Thompson SG, Ashton HA, Gao L, Buxton MJ, Scott RA. Final follow-up of the Multicentre Aneurysm Screening Study (MASS) randomized trial of abdominal aortic aneurysm screening. Br J Surg. 2012;99(12):1649-56.

2. Jacomelli J, Summers L, Stevenson A, Lees T, Earnshaw JJ. Impact of the first 5 years of a national abdominal aortic aneurysm screening programme. Br J Surg. 2016;103(9):1125-31.

3. Bown MJ, Sweeting MJ, Brown LC, Powell JT, Thompson SG. Surveillance intervals for small abdominal aortic aneurysms: a meta-analysis. Jama. 2013;309(8):806-13.

4. Glover MJ, Kim LG, Sweeting MJ, Thompson SG, Buxton MJ. Cost-effectiveness of the National Health Service Abdominal Aortic Aneurysm Screening Programme in England. Br J Surg. 2014;101(8):976-82.

5. Singh K, Jacobsen BK, Solberg S, Bønaa KH, Kumar S, Bajic R, et al. Intra- and interobserver variability in the measurements of abdominal aortic and common iliac artery diameter with computed tomography. The Tromsø study. Eur J Vasc Endovasc Surg. 2003;25(5):399-407.

6. Sidloff DA, Saratzis A, Sweeting MJ, Michaels J, Powell JT, Thompson SG, et al. Sex differences in mortality after abdominal aortic aneurysm repair in the UK. Br J Surg. 2017;104(12):1656-64.

7. Patel R, Sweeting MJ, Powell JT, Greenhalgh RM. Endovascular versus open repair of abdominal aortic aneurysm in 15-years' follow-up of the UK endovascular aneurysm repair trial 1 (EVAR trial 1): a randomised controlled trial. Lancet. 2016;388(10058):2366-74.

8. Comparative clinical effectiveness and cost effectiveness of endovascular strategy v open repair for ruptured abdominal aortic aneurysm: three year results of the IMPROVE randomised trial. Bmj. 2017;359:j4859.

9. Love-Koh J, Asaria M, Cookson R, Griffin S. The Social Distribution of Health: Estimating Quality-Adjusted Life Expectancy in England. Value Health. 2015;18(5):655-62.

10. Office of National Statistics. National Life Tables: England and Wales 2016-2018. 2018.
