# Supplementary material for: Modelling the impact of changes to abdominal aortic aneurysm screening and treatment services in England during the COVID-19 pandemic
Source: PLoS One. 2021 Jun 15;16(6):e0253327. doi: 10.1371/journal.pone.0253327 (PMC8205127; doi:10.1371/journal.pone.0253327)
Supplement: S2 Table — (DOCX) [file pone.0253327.s002.docx]

**S2 Tab**. **Predicted excess AAA deaths and emergency operations in the national surveillance cohort over 30y period**

| **Length of scan suspension** | **Scan suspension only**  **(S1)** | **+ 10% dropout/ annum for 1y (S2.1)** | **+10% dropout/ annum for 2y (S2.2)** | **+ 7cm threshold for 2y**  **(S3)** |
| --- | --- | --- | --- | --- |
|  |  |  |  |  |
| 6m | 2 (1) | 84 (44) | 152 (81) | 207 (111) |
| 12m | 9 (5) | 94 (49) | 163 (86) | 207 (111) |
| 24m | 40 22) | 123 (67) | 207 (111) | 207 (111) |
| 36m | 110 (61) | 191 (103) | 272 (146) | 272 (146) |
| 48m | 230 (126) | 305 (166) | 382 (206) | 383 (207) |
| 60m | 408 (222) | 477 (258) | 547 (295) | 548 (296) |

Notes: National surveillance cohort in March 2020: n =15,376; expected AAA deaths over 30y in NAAASP status quo = 2152; expected emergency operations over 30y in NAAASP status quo = 745.
