# Supplementary figures and images for: Modelling the impact of changes to abdominal aortic aneurysm screening and treatment services in England during the COVID-19 pandemic
Source: PLoS One. 2021 Jun 15;16(6):e0253327. doi: 10.1371/journal.pone.0253327 (PMC8205127; doi:10.1371/journal.pone.0253327)

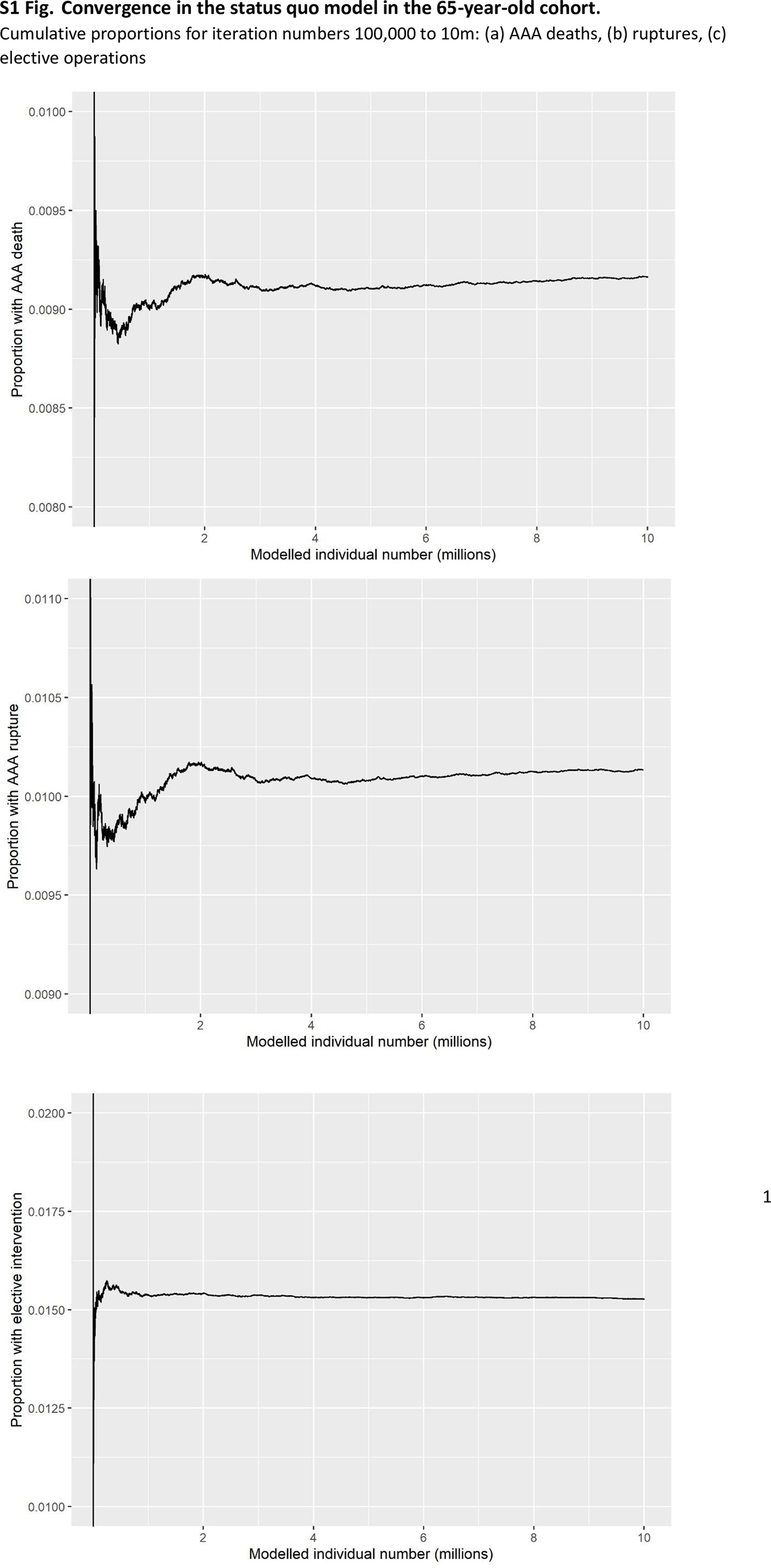

Supplement: S1 Fig — Cumulative proportions for iteration numbers 100,000 to 10m: (a) AAA deaths, (b) ruptures, (c) elective operations. (TIF) [file pone.0253327.s003.tif]

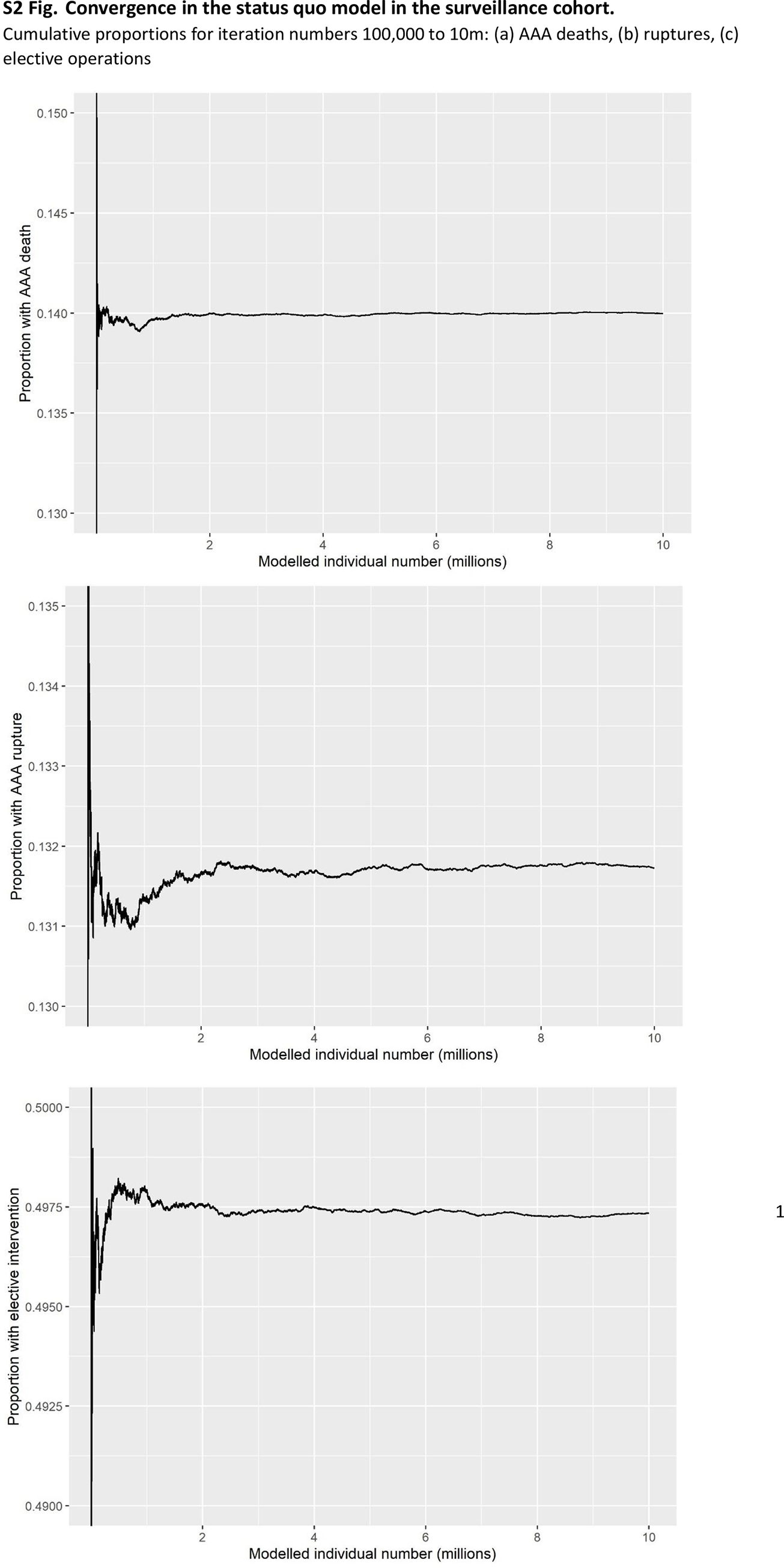

Supplement: S2 Fig — Cumulative proportions for iteration numbers 100,000 to 10m: (a) AAA deaths, (b) ruptures, (c) elective operations. (TIF) [file pone.0253327.s004.tif]

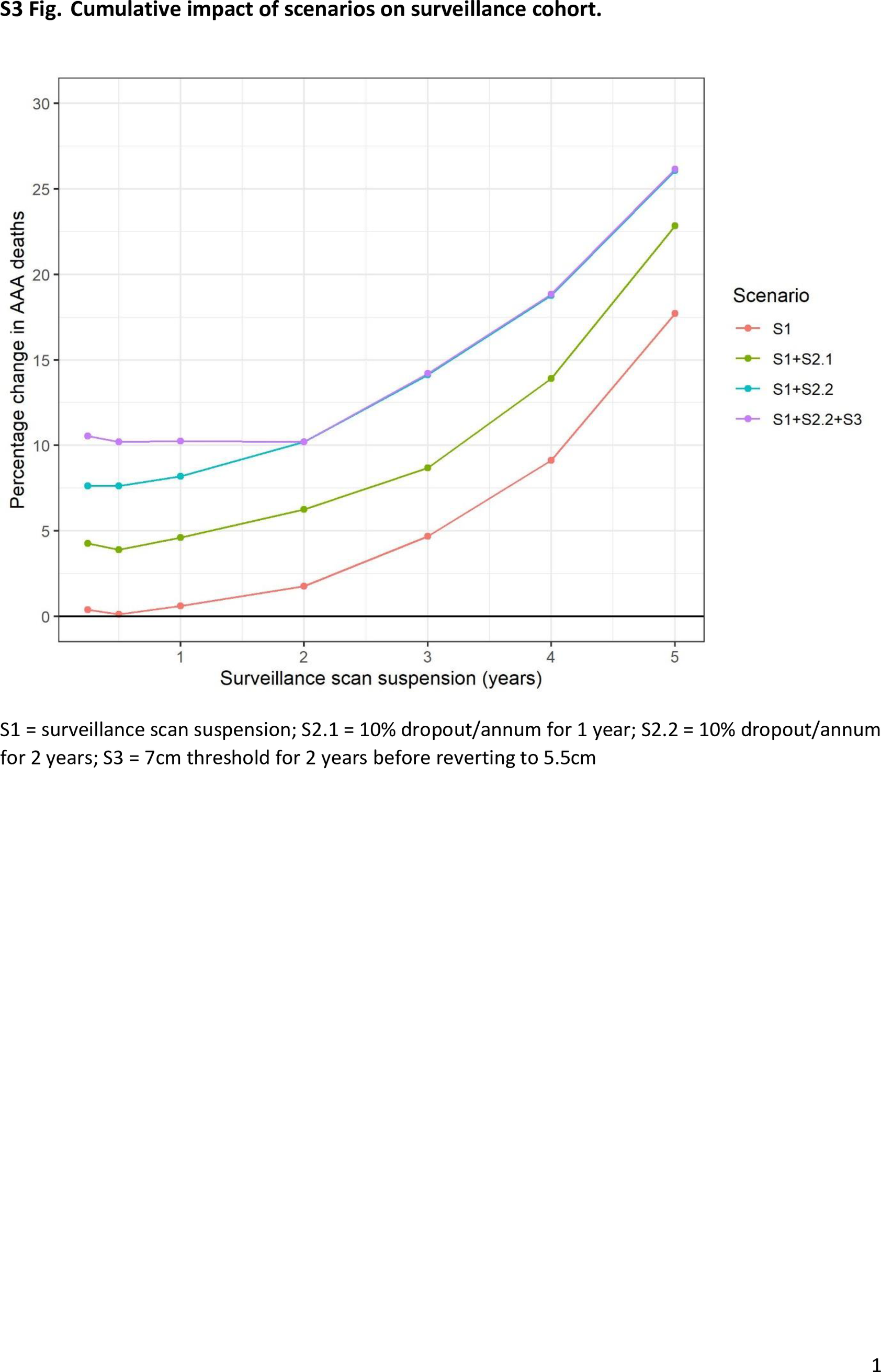

Supplement: S3 Fig — S1 = surveillance scan suspension; S2.1 = 10% dropout/annum for 1 year; S2.2 = 10% dropout/annum for 2 years; S3 = 7cm threshold for 2 years before reverting to 5.5cm. (TIF) [file pone.0253327.s005.tif]
