# Supplementary material for: Modelling the impact of changes to abdominal aortic aneurysm screening and treatment services in England during the COVID-19 pandemic
Source: PLoS One. 2021 Jun 15;16(6):e0253327. doi: 10.1371/journal.pone.0253327 (PMC8205127; doi:10.1371/journal.pone.0253327)
Supplement: Attachment — Submitted filename: Kim_PLOSOne_response to reviewers.docx [file pone.0253327.s006.docx]

**Modelling the impact of changes to Abdominal Aortic Aneurysm screening and treatment services in England during the COVID-19 pandemic**

*LG Kim^1^, MJ Sweeting^1,2^, M Armer^3^, J Jacomelli^3^, A Nasim^4^, SC Harrison^1^*

**PLOS Medicine: reviewer notes and author response**

Reviewer #1:

COVID-19 has forcibly enforced multiple changes in the health care system. The impact of these chances are to be seen in the coming years. The authors need to congratulated for taking effort to study one such impact using a previously described DES model.

I have no further comments on this article. The article can be accepted in the present format.

*We thank the reviewer for their support and positive feedback on our submission.*

Reviewer #2:

The authors are to be commended for recognizing the importance of event simulation modeling in this challenging patient population given the uncontrolled pandemic. The model they highlighted provides a robust approach in modeling outcome of patients with AAA and the emphasis is strongly suggestive on encouraging the recall for patients to attend surveillance screening program that is widespread in the UK. The authors repurposed a previously validated discrete event simulation model to investigate the impact of COVID19-related service disruption on key outcomes.

*We are pleased this reviewer recognises the importance of this work, and the benefits of having access to a well-validated, robust disease model to underpin our work.*

Broadly speaking, modeling tools are commonly used in practice to inform decisions on patients' treatment, risk prediction, and survival probability. This inherently depends on routinely collected data that provide estimates which in turn is dependent on modeling decisions focused on a certain population cohort. However, the decision-making should be supplemented with clinical judgment and evidence of additional risk factors. Thus, this represents an intricate issue coupled with multiple confounding biases to this event simulation mode discussed. The model used was not developed to tackle a wide array of profound variation faced in the current pandemic. Repurposing the model to match the current trend is pointless and is fraught with biases. I would have hoped that the authors have done a population study using shrewdness of clinical judgment made on CV19 versus non-CV19 affected patients and how this supplemented the clinical knowledge would have enhanced the fundamentals underpinning natural history AAA represents. I surely enjoyed the concept, but would strongly differ with the authors on the purpose of this study, its design, and the message they're intending to send across which does not produce anything meaningful unless the authors can present a validated product to match the current trend and pandemic.

*The reviewer here seems to be raising the point that COVID-19 may have an influence on the natural history of AAA. Whilst both short and long-term effects are not out of the question, there is currently no published evidence to support this view. We aim here to provide a decision-making tool at a critical turning point in the re-opening of AAA services, using the best available evidence at this time. Additionally, any impact of COVID-19 infection would also only apply to those who have had COVID-19; estimates of this range from around 10-20% of the population in the UK, though this varies widely by region and age-group.*


Reviewer #3:

This study uses a previously developed and validated discrete event simulation model for AAA screening to explore different approaches to post-lockdown service resumption.

Comments:

"The full pathway reflecting both the natural history and screening programme is described in detail elsewhere(8, 9). In brief, transitions between each state are modelled using rates and probabilities informed by NAAASP, and large randomised trials and studies of AAA screening and surgery (Supplementary Table 1)."
Can the authors please confirm if all parameters in the original model have been recalibrated with the latest data?

*The surveillance population used in this modelling exercise is informed by data relating to the relevant time-period in March 2020, as supplied by NAAASP (see lines 112-115). Our use of the DES model here does not include modelling of costs (which might require appropriate uplifting according to year). The parameters in question therefore relate to aspects such as AAA prevalence, growth and rupture. Although there may be some gradual changes in these elements over time (relating, for example, to changes in smoking habits in the screened population), the DES used was published in 2018, so it does not seem unreasonable to utilise these parameters in this work.*

"The repurposed DES model is extended here to allow events to be simulated from a cohort of individuals already under surveillance in the NAAASP, through simulation of key characteristics (age and aortic diameter) at the inception of the model ("time zero"), which is taken to be March 2020 when the initial UK national "lockdown" was imposed. The model is further extended to allow temporary suspension of a) invitation to primary screening, b) surveillance appointments, c) elective surgery, and to allow transitory increases in a) the AAA diameter threshold for elective surgery and b) non-attendance at both primary screening and surveillance scans (which may arise due to individuals engaging in more cautious behaviours as a result of the pandemic)."
Can the authors please present the formulation of the extended model, perhaps in the supplementary material?

*We have made the original and revised versions of the DES model available on github, and have now referenced this in the manuscript (lines #107-8).*

"We investigate the impact of changes relating to the invited group (varying suspension of invitation and attendance rates) and to the surveillance cohort (varying suspension of surveillance scans, drop-out rates, and time at increased size threshold for elective surgery) in a univariate manner. In addition, the cumulative impact of these changes in the surveillance cohort is also assessed"
In addition to these univariate analyses and combined surveillance scenarios, did the authors consider modelling various scenarios that combine both invited and surveillance models?
With this in mind, might there be transitions and interdependencies between the primary screening and surveillance cohorts that could be modelled?

*Since we are modelling a non-transmissible disease process here (AAA), there is no “interdependency” between cohorts; every hypothetical individual is independent in terms of their disease process and screening/management. Therefore, there is no problem with modelling separately the invited cohort who were not yet 65 in March 2020 and the surveillance cohort who were over 65 with known AAAs at this time. The total number of AAA deaths for the national population (invited and surveillance cohorts combined) for example, can be reasonably calculated by adding the two separate results together. Finally, although the requirement for access to common resources (such as scanning and surgical facilities) in both cohorts could be described as an interaction between the groups, we do not model limited resource use here and thus assume no delays resulting from backlogs. We discuss this limitation in lines #255-259.*
